# Chicago Gynaecological Society: Meeting of January 21st

**Published:** 1887-03

**Authors:** W. W. Jaggard

**Affiliations:** 2330 Indiana Avenue


### SOCIETY REPORTS.

---

TRANSACTIONS OF THE CHICAGO GYNÆCOLOGICAL SOCIETY.

*Regular meeting, Friday, January 21, 1887.*

*I.*—PARKES. *A Case of Interstitial Pregnancy, With Removal of the Product of Conception Through the Uterine Cavity.*

*II.*—BARTLETT. *A Case of Obstetrics, with Remarks.*

*III.*—ETHERIDGE. *Antiseptic Tamponnement of the Vagina in the Treatment of Pelvic Inflammations.*

The PRESIDENT, CHARLES WARRINGTON EARLE, M.D., in the chair.

I.—PROFESSOR Charles T. Parkes made the following remarks upon:

A CASE OF INTERSTITIAL PREGNANCY, WITH REMOVAL OF THE PRODUCT OF CONCEPTION THROUGH THE UTERINE CAVITY, with the exhibition of the specimen.

The specimen, which I exhibit to-night, comes from a case which has been of extreme interest to me, and is, I

think, the remains of a conception, which was certainly outside of the uterine cavity, and which I succeeded finally in delivering through the womb. It was taken from a lady, 33 years of age, who, seven years ago, was delivered of a child at full term. The child is now living. A year after that, she was taken with hemorrhage and had quite a severe bleeding, every month or second month, for two years. Some time after her pregnancy, she was operated upon for laceration of the cervix, but the operation had little effect upon the hemorrhage. Two years ago she again became pregnant, and was delivered at the proper time. This child is still living. The lady came under my charge last September for hemorrhage from the uterus. On examination I found a globular mass in the lower portion of the abdomen, as large as two fists, very hard and tense. When I felt it through the abdominal walls, my impression was that it was a fibroid growth. Upon digital examination, I found the cervix dilated sufficiently to admit the finger very readily, which went over the surface of a smooth mass in the uterine cavity. This led me to think that it was a fibroid tumor with a broad base, probably a submucous tumor, which gave rise to the hemorrhage. On that supposition I placed her on the ergot treatment and kept it up for a week, twenty drops of the fluid extract every six hours. This gave rise to such severe attacks of pain that the patient could not bear the treatment any longer, but it had the effect of diminishing the flow of blood and increasing the dilatation of the cervix. I took pity upon her on account of the pain and gave a couple of hypodermic injections of morphine, when the pain ceased, the cervix began to contract again and soon reached its normal size, and the patient recovered from the acuteness of the disturbance, but the hemorrhage still continued, accompanied with a flow of muco-pus. I attended her from the 16th of September until the 14th of October; as she was getting along pretty well, I supposed the action of the ergot would gradually force this mass down so that it could be removed. My visits ceased and I heard nothing more from the patient, except an occasional report that she was getting along in the same way, until the 10th of December, when her husband came into my office and showed me a little piece of bone, or a piece of hard substance that looked like bone, which he said his wife had picked off the napkin. It had the appearance of fetal cranial bone. He asked me what it meant, and I told him I could not say, but would see his wife. On inquiry, I found that the flow of blood had ceased, but the flow of pus had increased, and occasionally there was extruded a piece of this bony substance. On digital examination, I discovered the os and cervix filled with particles of this bony substance, and after removing them, I found it impossible to introduce my finger into the cervix. The external tumor was reduced considerably in size, and was low down in the pelvis, and could be felt projecting through the anterior vaginal wall. I then decided to dilate the cervix. I introduced as many tupelo tents as I could get into the cervix—at first but two of fair size—to their full length, and allowed them to remain there over night, when I removed them and introduced four more. That evening I removed them, and the cervix was dilated so that I could easily introduce my finger. As I had examined the uterus with the sound at my first visit, and it went around this mass to its full length, I supposed I had nothing but a fibroid to deal with. When I had dilated the cervix with these tupelo tents, I found I could not get at the mass of the growth, my finger going into the cavity of the uterus. At the distance of one joint and a half inside the cervix, I found a little opening, and projecting through this opening—about as large as the end of a pencil—were some of these particles of bone. Then the query arose, how was I to get into this cavity, and what was it ? a double uterus, with multiple pregnancy at the last conception—one delivered and the other retained ? I was at a loss to know what it was. (But finally concluded it to be an intra-mural pregnancy.) I had the particles examined under the microscope, and they showed the structure of fetal bone. Then I thought of using the tents, to increase the dilatation, but was troubled with the fear that I should have a severe scepticemia come on as soon as this outside cavity was opened to air. But I was convinced that unless I tried to do something, the patient would pass out of my hands ; so I decided to keep on with dilatation. On the 20th of December I began introducing the tents, and within two or three days after their removal, the cervix was again contracted so that it would not admit the finger. I introduced the tents again, and met the same difficulty in exposing the mass. The thought struck me that if I could not get the large body out of the small opening, I could diminish the size of the mass ; so I introduced small forceps into this opening, and took it away piecemeal. All this time I had the entire uterus under my command, because it was an easy matter to bring the cervix down to the vulvar orifice. On the 24th I introduced tents and dilated it, so that I could introduce two fingers very readily, and finally got one of my fingers into the opening in which this body (indicating the specimen) was found. I then began to separate it and pull it away, getting hold of it with strong forceps. Sometimes I succeeded in bringing away a large mass of flesh, which looked exactly like that from a macerated fœtus, the skin macerated and parchment-like. This was continued up to the 30th. Passing over the New Year, and allowing the patient to rest without interference, on January 9th I introduced four tupelo tents, a little longer than the ordinary, and fortunately succeeded in getting one or two into the opening in which the body was found; so when I removed them that evening, I was enabled to bring away the entire mass and pass the finger into the cavity afterwards. It was very irregular, as though the uterine tissues had been forced into the irregularities of the foreign body. Since that time the patient has improved, the bleeding has ceased, the uterus has diminished in size, and she is up and about the house. I have had all parts of this specimen examined under the microscope. The fleshy mass shows connective tissue, muscular fibres, blood-vessels and hairs. The osseous material shows all the characteristics of fœtal bone.

DISCUSSION.

THE PRESIDENT: Was there a history that would lead you to suppose, that at any time during her invalidism there was a pregnancy, or a pelvic hematocele, or anything of that kind?

PROFESSOR PARKES: At the time of her last pregnancy, she was very large and yet was delivered of a child that weighed but six pounds. Her abdomen was very large for some time after the delivery of this child. Again, there is a history several years back, of a period when menstruation ceased, and she supposed she was pregnant, but nothing came of it.

PROESSOR W. W. JAGGARD said the interesting specimen presented by Professor Parkes was a typical lithopædion—calcareous capsule, containing the fœtal structures infiltrated with lime salts.

He thought the diagnosis of interstitial pregnancy highly probable. It was impossible to make a positive diagnosis without a post-mortem examination of the maternal organs. Carl Braun (*Lehrb d. g. Gynaek*, 1881, p. 128) was responsible for the statement that the formation of a lithopædion occurred only in case of extra-uterine pregnancy. Spiegelberg (*Lehrb. d. Geburtshülfe*, 1882, p. 342) however, indicates that this proposition is too general. The formation of a *uterine lithopœdion* occurs infrequently in the human female, but is not unusual in ewes. Koeberle (*Gaz. hebd.* No. 34, 1866) extracted by abdominal section a lithopædion from the rudimentary horn of a *uterus bicornis.*

The formation of a lithopædion, therefore, was not a reliable sign in the differential diagnosis between uterine and extra-uterine pregnancy.

---

II.—DR. JOHN BARTLETT read a paper, entitled,

*A Case of Obstetrics, with Remarks.*

Recently I was requested to assist a younger physician in a case of midwifery. Dr. H. had been called some hours before my coming. He found a healthy, well-built woman in labor with her eighth child. Hitherto she had had no difficulty in her confinements. She had been in labor some hours, and, although the pains were very strong, the os fully dilated, and the head presenting, no progress had been made. A midwife had been in attendance. The Doctor attempted to use Elliot's forceps, but, because of the high and abnormal position of the head above the pelvic brim, he had desisted from his purpose. Upon examination, I found the os widely dilated, the crown of the head presenting. By introducing the hand into the vagina, my fingers, directed toward the left sacro-iliac synchondrosis, encountered and passed slightly beyond an extremity of the head-ovoid which I supposed to be the occipital protuberance, but near it was so distinct a fontanelle as to lead me to examine the opposite extremity of the head. Passing the hand deeply behind the left foramen ovale and well above the pubes, the fingers embraced the occiput; sweeping well backward again over the side of the head they traversed the temporal region till the ear was reached and carefully outlined. Still farther backward the fingers passed over the frontal eminences, which had at first been mistaken for the occipital protuberance. The head was floating above the pelvic brim; the frontal region sinking somewhat below the plane of the superior strait. The crown of the head rested gently upon the pubes, while the occiput rested so far forward over the pubic bones as to be distinctly appreciable to sight and touch from without. Having determined the position of the head, I proceeded to inquire the cause of its detention; for it did not impinge with force upon any portion of the circumference of the brim. Passing the fingers along the side of the head I felt for the cord around the neck. A coil of cord was immediately encountered and pressing a little farther upward a second, third and fourth coil were detected. I felt authorized to announce to Dr. H., as the cause of the dystocia, the suspension of the head above the brim by the cord shortened by four coils about the neck, The fingers were passed about the occiput and it was pressed downward and backward, throwing the forehead backward and upward above the brim, and bringing the occiput slightly into the pelvis, the pains meanwhile having a decided effect in assisting the manœuvre. I now intended to seize the occiput with the vectis, and so deliver. Upon trial with the fingers, however, I appreciated that, as the occiput had descended into the pelvis, the forehead had risen above it, so that the power of the vectis as a tractor would not be available, seeing that the forehead, the opposite end of the lever I proposed to move, was not fixed but floating, the fœtus yet resting with the equators of the head well above the brim. As a lever to keep the occiput in proper relation to the inlet, the "Roonhuysen" would have served admirably; but in order to make available traction upon the head it would be necessary to lay hold of it with the forceps.

Accordingly, with very little trouble, though locking was effected within the vagina, the head was seized with a well-curved Simpson's forceps, and readily brought down. The expectation was, as soon as the head was delivered, to place quickly two clamp forceps on the cord and cut it between these, in order to escape the embarrassment which the several coils about the neck might occasion. The first loop however, was easily drawn over the head, the other coils were then readily released. The child which weighed 11 pounds breathed at once, seeming but little affected by the unnatural position of the funis. The length of the cord was 46 inches.

It may be considered what other line of practice might have been pursued. It might have been practicable to disengage the cord from the neck and in this way remove the cause of the dystocia. To this practice was the serious objection that with the head floating above the brim, the liberation of such a length of cord so near the pelvic inlet, might have led to its prolapse.

"The question as to the shortness of the cords being a cause of dystocia," says Joulin, "has passed through a variety of phases before our day". "Accepted by all accoucheurs from Mauriceau to Baudelocque as quite a frequent obstacle to the termination of labor, it was first rejected almost entirely by Baudelocque; and Gardien, still more radical, did not admit it at all. Lachappelle and Duges were but little disposed to consider it as a cause. Finally Desormeaux, Maygrier, Velpeau and Moreau, while they admit shortness of the cord as a cause of dystocia regard it as very rare; and this view is generally accepted to day." Some of the recent standard writers omit to mention a shortened cord as a cause of difficult labor. Spiegelberg states that too short a cord can only interfere with the advance of the child in the lower part of the parturient canal. Cazeaux, on the contrary, says that a shortening of the cord may retard the progress of the head at the superior strait. He writes: "We have met with a case in which unusual shortness of the cord, which was only nine inches in length, certainly detained the head above the superior strait for fifteen hours after the rupture of the ovum, and the entire dilatation of the os uteri." Among more recent writers, Lusk has a chapter on shortness of the cord. He reports the case of a neglected primipara, whom he assisted with the forceps after she had been in ineffectual labor for five days. "Her temperature was 103.5° F. she was in great agony. The external organs were inflamed. She died on the fifth day, the external genitals becoming gangrenous. The cord, tense and coiled a number of times around the neck, was the cause of the delay."

I cite two cases of dystocia from the cord encircling the neck. The first from that worthy man-midwife, Guilleaume de la Motte, showing to what heroic expedients resort was had in these cases before the forceps came into use. The other case, from Smellie, is so similar in method of diagnosis and treatment to the one just reported, that it can hardly fail to interest.

LaMotte writes: "A young woman of this town, big with her first child, who had been very healthy during her pregnancy, found herself attacked with slight pains, that soon became very sharp and pressing. I was called to her in a hurry on the thirteenth of November 1697. I found the waters come away, and the child well situated. As the pains were brisk and frequent I thought the work would soon be over, but though the child kept moving continuously, was well situated, and far advanced, it remained six hours at the crowning. I was well assured that nothing but the cord could keep it there so long, when the pains were so excessive, but I saw no way to help her, there being no room to pass the fingers nor even the nail, between the head and bottom of the vagina, except toward the lower part where I slipped my finger dipped in oil as far as the chin, which I brought forward little by little, and then the head also; and kept pushing on my finger notwithstanding the sharpness of the pains. I reached at last the neck which I found entangled with the cord. I introduced my finger between them and slid the scissors upon it, with the button end toward the neck, and cut the cord through; the child came out immediately, asphyxiated but living; it kept groaning for two hours, and then did very well, except that it remained dumb. Whether there was anything displaced in the organs of speech, or the recurrent nerve obstructed, I know not. The cord had three turns; I cut only the last, which was that next to the placenta."

"In June, 1751," says Smellie, "I was called by a midwife to a woman who had been many hours in labor and found that after the discharge of the waters, the head was forced low down by every pain, but afterwards drawn up again. I was likewise informed that formerly she used to have large children and quick labors.

"Encouraged by this intimation, I tried to turn the child, but was prevented by the strong contraction of the uterus; but in making this trial, and raising the head I not only found the funis surrounding the neck, but likewise the uterus contracted before the shoulders. This last I dilated with my fingers as much as possible, then withdrawing my hand, applied the forceps and delivered the child, which had been dead for some days. The funis was three times around its neck being much tumefied and of a livid color."

In connection with the case reported by myself, I propose to make some comments upon the mode of determining the position of the head in labor. From time immemorial, it has been the custom of teachers to describe with particularity how the position of the head may be determined by the tips of the fingers by means of the sutures and fontanelles.

Whatever skill or tact others may be endowed with, or may have acquired in such methods, for myself I wish emphatically to declare that such examinations are often entirely insufficient to furnish me with the desired information; and that now, after years of careful observation, I am not unfrequently at a loss to determine the position of the head after the usual examination *per vaginam*, and that I am occasionally led into an error in this regard only to be dissipated by the birth of the head. Nor am I alone in this want of capacity; a number of experienced obstetricians, with whom I have conversed on this subject, have expressed like uncertainty in determining the position of the head by the means mentioned.

The veteran John S. Clark, the most experienced practitioner in obstetrics that I have ever met, has on several occasions denounced the directions above referred to and so often repeated in the text-books as a delusion and a snare. The late Dr. Grosbeck, after fifty years of obstetrical practice, declared that he never could rely upon determining the position of the head by the methods under consideration. And the painstaking, accurate and deliberate surgeon, Dr. R. G. Bogue, does not boast of much better success. One of the most learned obstetricians in this city, an able lecturer on midwifery, once assured me that while he repeated fluently enough to his classes the stereotyped methods of determining the position of the head by the fontanelles and sutures, he often found, as the head passed the vulva, that the "data" furnished by the tips of the fingers had led him into gross error. While, in many cases the position of the head may be easily and certainly recognized by the ordinary methods, it is yet certain that in other instances, more especially when difficulties make a knowledge of the head's position particularly desirable, nothing positive as to its attitude can be made out by the average practitioner, by feeling in the usual way for sutures and fontanelles.

Nor is this appreciation of the difficulty of determining the head's situation new. That admirable obstetrician, William Smellie, who was one of the first to appreciate the desirability of knowing the head's position, and who perhaps earlier than any other accoucheur taught how such knowledge could be acquired, was often foiled in his efforts to ascertain the head's true situation. He writes in his "Observations" as follows: "The head though low down was so swelled that I could not distinguish its position, for I could feel neither suture, ear, nor back part of the head." And in another place he writes, "I could not in any way, by the sutures or otherwise, distinguish the right situation of the head. I introduced the forceps at random by the sides of the pelvis." And again, "The head was so large and compressed into such a lengthened form that I could not push up my finger at the pubes to feel the ear or neck; neither could I distinguish the situation of the head by the sutures, because the scalp was so swelled; nor could I move the head upwards in order to feel the upper parts, such as the ear, neck or face." And also, "I felt something like the vertex down at the lower part of the pelvis, but we were all mistaken as to the position of the head. I thought the forehead toward the sacrum. I mistook the posterior for the anterior fontanelle. I was surprised to see the (supposed) occiput come along under the pubes, not with hair, but bald and smooth. We had all been mistaken as to the position."

How then in cases requiring a knowledge of the head's position is such information to be obtained? I know no better way of answering this question than by making reference to the practice of Smellie. Please to note the thorough methods by which he satisfied himself of the size or position of the head in the several cases here cited. "I knew the child was small because I passed my finger all around the head." And, "I perceived that the head was not large, because I could easily introduce my finger all around the lower part of it." Desiring to ascertain the position he says, "I scooped up the head above the brim of the pelvis, and as I slipped my hand flattened between the sacrum and the child's head, I felt with my fingers the back part of the neck" (determining the position of the occiput). And again, "I turned the back of my hand down towards the sacrum and raised or scooped the head gently to the upper part of the pelvis; and now with my fingers I felt the posterior part of the neck, and distinguished that the pelvis was not distorted. Thus informed, I introduced the blade of the forceps," etc. In reference to another case he says, "Being foiled in delivering the head, which was not large, after having properly applied the forceps I disengaged the instrument, and raising the head again (out of the pelvis) and found the difficulty was owing to the left shoulder being over the pubes. I got hold of the arm, brought it down, and again fixed the forceps and delivered, pulling gently at the hand."

From these extracts it will be seen that Dr. Smellie did not content himself with vaguely touching such portions of the presenting part as might be reached by the introduction of one or two fingers, but that he introduced deeply the half hand, or the whole hand, and passed the fingers into every available space; not hesitating, when necessary and practicable, to lift the head above the brim that he might get his fingers about its salient points as the ear, the face, the back of the neck. It is noteworthy that it is only when circumstances prevent the head being thus "traced," that Smellie recommends that "the observation" be taken from the fontanelles and sutures. In the case which is the basis of this paper, the vaginal examination was made after Smellie's method. The steps of the procedure have been given in detail with the purpose of illustrating his teachings.

DISCUSSION.

DR. PHILIP ADOLPHUS: The diagnosis of the position of the child in head presentations by means of sutures and fontanelles is not as difficult to the physician who has been in attendance during a case of labor as has been stated this evening. The gradual descent of the head into the pelvis will permit the recognition of the landmarks by *repeated* examination with the finger.

In diagnostic obstetric investigations, palpation of the abdomen, the examination of the child's head and the pelvis of the mother by bi-manual palpation, should be conducted on the same principles as in gynæcological cases. An empty bladder is also essential to a successful diagnosis.

Such an examination will insure the recognition of the position of the child's head, and other necessary information.

The consulting physician, who encounters a tender abdomen, tumified soft parts and a swelled scalp in an exhausted patient, has a far more difficult task. The same rules, together with the introduction of the hand as far up as is required, under anæsthesia, will give him the necessary information.

The experience of the eminent writer of this paper, as well as that of others, shows plainly that a refinement of diagnosis is not absolutely essential. Many cases of labor are completed, in which the diagnosis of the position of the head has not been ascertained by its sutures and fontanelles. Moreover, cases which require delivery by forceps are frequently skillfully handled when the operator has not been enabled to ascertain the position of the head. We explain this by stating that the mechanical adaptation of the child's head to the bones of the pelvis is perfect; sooner or later the child's head, if

*not disproportionate in size to the pelvis*, will accommodate itself to its configuration, provided other obstacles in its path have been removed by the attendant. We state, also, that the position of the head does not determine the position of the blades of the forceps, but the position of the blades is always determined by the anatomy of the mother. Therefore, the forceps should be applied along the sides of the pelvis, and its pelvic curves should correspond to the curved axis of the pelvis.

Its introduction is governed by the direction of the obstetric canal, the globular head of the child, and the cranial and pelvic curves of the instrument. The direction of the obstetric canal in a woman in labor is not the osseous pelvis merely, but the pelvis covered with soft parts, whose terminal outlet is not at the point of the coccyx, but at the anterior commissure of a greatly distended perineum, a distance of ten to twelve inches during labor.

The blade of a long double-curved forceps—having both the cephalic and the pelvic curves—is guided into the pelvis by the fingers, and insinuates itself between the head and the soft parts of the mother. To facilitate the introduction of the second blade the first blade is gently elevated and rotated as much in a lateral direction as possible. The same manipulation is repeated with the second blade. In many cases the elevation of the blades and their gradual rotations for the purpose of locking them, adjust the blades of the forceps to the head of the fœtus, as they have already adjusted themselves to the mother's pelvis; and now traction, some compression, and slight leverage (if necessary) complete the delivery of the child, *which will rotate spontaneously within its blades* during traction, owing to the anterior and posterior planes on either side of the cavity, and the resistance of the floor of the pelvis.

It is best that the exact position of the head should be known, but such knowledge is not essential to its safe extraction; on the contrary, it is not correct to apply the forceps to the sides of the fœtal head when its position is oblique or transverse, for if its pelvic curves are twisted, injury must be inflicted on the mother.

PROFESSOR W.W. JAGGARD: I have listened to the reading of Dr. Bartlett's scholarly paper with interest and pleasure. His allusions to the wisdom of the ancients are always timely and judicious, notwithstanding the fact that, in general, the results of modern observation and experience are entitled to a higher degree of consideration. I hope to be pardoned for making one or two criticisms.

The diagnosis of dystocia, by reason of a short cord, is not adequately established by the clinical history of the case. The ease with which the vertex engaged, after manipulation, and descended, after application of the forceps, the absolute length of the cord, 46 inches—even with four loose coils around the neck, not relatively short—the condition of the child when born, these are facts which do not indicate that the length of the cord consituted a mechanical hindrance to the progress of labor. The author has quoted Spiegelberg, who is of the opinion that shortness of the cord constitutes a mechanical hindrance only when the presenting part reaches the lower portion of the parturient canal. The only method of determining with certainty, in the concrete case, that shortness of the cord is acting as a mechanical obstacle, consists in the introduction of the fingers, direct contact with the cord, and the detection of the abnormal tension. If the case related by Dr. Bartlett was one of dystocia, and if the "occiput projected so far forward over the pubic bones as to be distinctly appreciable to sight and touch from without," does it not seem a plausible hypothesis that the child was presenting slightly obliquely, and that the operator performed cephalic version? "The fingers were passed about the occciput and it was pressed downward and backward, throwing the forehead backward and upward above the brim, and bringing the occiput slightly into the pelvis, the pains meanwhile having a decided effect in assisting the manœuvre."

In treating of obstetrical diagnosis, in general, Dr. Bartlett does not mention the signs derived from inspection, auscultation, and particularly *abdominal palpation*. I am induced to call attention to this topic for the reason that, notwithstanding the writings of Kucher, Mundé and Richardson, the recognition of the value of abdominal palpation in obstetrical diagnosis in the best recent text-books, and the translation of Pinard's Treatise, by Dr. L. E. Neale, of Baltimore, still many practitioners affect to disregard the paramount importance of the method. Litzmann (1865), Halbertsma (1870), Winckel (1878), Credé (*Gesunde und kranke Wöchnerinnen*, Leipzig, 1886, p. 80, *et seq.*), in order to prevent the infection of parturient women in their respective lying-in hospitals, have omitted all examinations *per vaginam* for months at a time, with most gratifying results. Under these conditions, external examination has proved perfectly adequate in the diagnosis of presentation and position.

I confess to a feeling of decided surprise upon hearing that a medical man, with the average degree of tactile sensibility and even moderate experience, should necessarily have difficulty in the diagnosis of position, by indagation, in normal, vertex presentations, after engagement, before the formation of the *caput succedaneum*,—the *os externum* being dilated or dilatable, the bag of waters, intact or ruptured. I am under the impression that failure to make an accurate diagnosis, by examination *per vaginam*, under the conditions specified, is due in very many cases to inattention. It is an obstetrical maxim of importance that both fontanelles and their sutures should be felt before making a diagnosis, when vaginal touch is exclusively employed. When an extensive *caput succedaneum* has formed, or ossification of the fœtal skull is advanced, or in case of subnormal tactile sensibility on the part of the accoucheur, no absolute contra-indication to the introduction of the half hand exists. In forceps cases, a correct diagnosis of the position of the vertex must be made, since that instrument ought to be applied first with reference to the pelvic walls, and then adapted to the child's head, before the exercise of its most important—and as I believe, exclusive—function of traction.

DR. EDWARD WARREN SAWYER: I wish to speak of an interesting experience which occurred to me. A gentleman who had carefully translated the book alluded to called me in consultation to assist him. He had, by means of bimanual palpation, diagnosticated a presentation of the vertex, but on my examination I found the buttocks were presenting. I think in most cases abdominal palpation is of no service whatever to the majority of practitioners. I have experienced the same difficulty that Dr. Bartlett has so graphically described in recognizing the position of the head by the introduction of the finger into the vagina. And after a long practice, so uncertain am I concerning the position that I never think of applying forceps until I have introduced enough of my hand to recognize some part of the face or head, in order to determine the exact position of the head.

DR. J. SUYDAM KNOX: In regard to making an exact diagnosis of vertex positions, I must often confess failure if I rely only on digital touch.

I have no doubt if the practitioner is called early to a case of labor before the uterus has become contracted, and the bag of waters has been ruptured, that it is possible by abdominal palpation to make out the position of the fœtus. When, however, labor has gone on for several hours the uterus becomes irritable, contraction and retraction taken place, and the liquor amnii to some extent discharged, I am satisfied that it is often impossible to make out a diagnosis of the position of the fœtus by digital examination. Even if you can determine that the vertex is presenting, you cannot then make out the position exactly. I have no doubt that Dr. Jaggard is correct about those large obstetrical hospitals in Europe. The diagnosis is made because the patient is under observation from the time labor begins. But the busy practitioner is called after labor has progressed some hours, and the uterus is so irritable that as soon as he begins to make any abdominal examination it contracts, and it is impossible easily to make a diagnosis. I do not introduce the hand into the vagina in many cases, but when the labor is protracted, and I think the use of forceps necessary, and I cannot make out the exact position of the head, I give the patient an anæsthetic, and introduce the hand sufficiently to find out how the head lies. I cannot see how sepsis can occur by the introduction of that portion of the hand necessary to make a diagnosis, and I think the diagnosis should be made before instruments are applied. I have several times tried the oblique introduction of the long forceps, but doubt the wisdom of introducing them obliquely without reference to the shape of the mother's pelvis and attempting traction. It is much better to apply the forceps to bring down the head, with reference only to the maternal passages, and when the head has been brought through the superior strait, to unlock the forceps and allow rotation before effecting delivery. At times it is better to remove the forceps entirely, and to re-apply them after rotation has occurred.

PROFESSOR DE LASKIE MILLER: I was much interested in the very lucid paper that has been read. It is true that there were many statements that seemed strange to me. The paper was on the treatment of complications resulting from short cord, and the illustration was a case in which the cord was 46 inches long with only four coils around the neck. This should not, it seems to me, be a cause of dystocia; but admitting that it was, we come to cases of actually short cord causing dystocia. Take a cord that measures only four inches in length, or a case of labor which has occurred in which there is no cord, of course there must be a placenta, and the fœtus is attached through this directly to the wall of the uterus. In such a case how can delivery take place without applying traction force sufficient to sever the placenta? Physiologically the contraction of the uterus, especially after dilatation is completed, is attended with a muscular retraction of the fibers of the body and fundus, which diminishes the cavity of the uterus and has the effect of severing its relation with the placenta. It is therefore possible for the placenta to be severed from its attachment to the uterus, by this retraction, and moreover there can be no injury to the uterus from the short cord if the contractions are normal, for while the organ is contracted the relation between the attachment of the cord and the uterus, or the placenta and its attachment to the fœtus, is not extended, rather shortened, so that the advance of the child can take place and delivery result.

A case is brought to my mind, which occurred in my own practice, of a primipara who was perfectly healthy, with nothing abnormal until about the time labor commenced. When I saw the patient in the first stage of labor, she remarked to me that she had felt no movement of the child for a considerable time, but this produced no impression on my mind, for it is a common thing for patients to say, and I paid no attention to it. The labor proceeded, and as the head was expelled from the vulva, I did as I always do, passed the finger instantly to the neck with the view of searching for the cord, and if it is found there, liberate it. I found two or three coils of the cord around the neck, and they were so tightly drawn that it was impossible to disengage them. In order to deliver the child readily, I severed the cord. I noticed there was no circulation, and the child was still-born, past all possibility of resuscitation, and it had been dead a long time, for I found a knot in the cord drawn so tightly that the circulation was entirely cut off. In addition to the coils around the neck the cord passed over the shoulder, under the opposite arm, around the body and under the knee, and possibly there were other coils. It appeared very much like the statue we see of Laocoön. I infer that the movements of the fœtus at the time labor commenced or shortly before had tightened the cord, causing its death. This is the only case I have met in my practice in which I could satisfactorily trace the death to the closing of the knot. In regard to diagnosis of position, I was not aware that it is so difficult to make the diagnosis of position. I believe the practitioner should make out a diagnosis by abdominal palpation, which can be done with great facility if he is accustomed to the practice; but I also believe that the diagnosis can be made with one fontanelle and the sutures. We can certainly discriminate between the anterior and posterior fontanelles.

DR. JOHN BARTLETT: A Fellow has expressed surprise that as a means of diagnosis I have not made reference to abdominal palpation. I purposely limited my remarks to the ordinary methods of vaginal examination. I may, however, give it as my opinion that the method of determining the position of the head by abdominal palpation will probably prove available to those only who are capable of diagnosing head positions by the ordinary examinations per vaginam.

Doctors Jaggard and Miller have called in question the assigned cause of dystocia. To them it does not seem probable that the shortened cord was the cause of delay. Their objections are well taken. In this case there are two facts which give rise to the question whether the cause of the dystocia was really the shortening of the cord, the one in itself offering at first glance a sufficient cause for delayed labor, viz., that the head was projecting decidedly forward over the pubic bones; the other seeming to guarantee freedom from restraining tension on the part of the cord, namely, its unusual length; so that after the delivery of the head the funis, though shortened, was not too tense to admit of its coils being released in the usual way. It must be considered, however, in reference to the abnormal position of the head at the superior strait, that while its attitude presented an impediment to the descent of the occiput, it invited a facile descent of the forehead; and yet this descent did not occur. Besides, the head could be swayed to and fro in the median plane of the occipito-frontal diameter so easily and freely as to give the impression that it swung on a pivot at the neck. In fact, it was this sensation imparted to the hand that suggested the probable suspension of the child by the cord; and this suggestion was strengthened by the apparent absence of any natural tendency of the head to settle into the excavation, either in the first instance as a brow and face presentation, or subsequently, as a right occipito-anterior position.

In regard to absence of great tension of the cord after the birth of the head, it is to be considered that without calling in question the possible detachment of the after-birth, surging of the coils about the neck, etc., the well-known mechanical principles by which the attached placenta in such cases in some measure keeps pace, so to speak, with the descending head, so clearly described by Dr. Miller just now, may themselves offer an answer to the objection that the cord was not found more tense after the head was delivered. No argument, however, can place the case certainly within the category of those in which dystocia is due to shortening of the cord. It will be perceived that I have regarded the case as interesting rather because of the unusual diagnosis of the malposition of the funis than as one in which this abnormity produced dystocia; and that I have availed myself of the free exploration of the presenting part by which the diagnosis was made as an opportunity to present what I regard as the more important part of this paper. I refer to my views as to the insufficiency of ordinary vaginal examinations as means of determining presentations and positions in labor. Upon the discovery of the four coils of cord about the neck, in association with other circumstances and conditions mentioned, I conceived the circling of the funis to be the cause of the dystocia, and conducted the delivery in accordance with that idea.

Critcisms upon the plan adopted should be made in this case, as in others, from the ante-partum standpoint of information. They should not be based, for instance, upon the knowledge that the cord was of very unusual length. This surplus in the cord's length threw a new and unexpected light upon the case, casting difficulties, before prominent in the foreground, into shade, and causing possibilities not before visible distinctly to appear. In this new light an opinion might be formed that the case left to nature would have terminated well, and that all interference was unnecessary. And yet I incline to the opinion that the ante-partum view of the case through the dark glass of the clinical obstetrician was the correct one.

III.—DR. JAMES H. ETHERIDGE made the following remarks upon

*Antiseptic Tamponnement of the Vagina in the Treatment of Pelvic Inflammations.*

What I have to present refers to tamponnement of the vagina and supporting the uterus in cases of pelvic trouble, notably of inflammation and enlargement of the uterus, and as the work has grown upon me, other complications in the way of pelvic trouble have also been treated with a result that has rather surprised me. For it I claim nothing original. A year ago last fall, I commenced the treatment of a case of general metritis and prolapsus of both ovaries with enlargement, which had brought the woman to a very low state. She had had all sorts of operations performed and advised,—repair of the neck of the uterus and the perineum for laceration,—and had been recommended to have the ovaries removed. Upon examination I found the uterus immovable. I placed the woman in the genu-pectoral position and tamponned the vagina with absorbent cotton, saturated with boro-glyceride. The vagina was douched before the tampon was reapplied, and of course everything was removed during menstruation. This was kept up for three months and she began to have less neuralgia, which had made her life miserable. I kept up the treatment three or four months longer, when there was complete mobility of the uterus, and she went out of doors and to church. The neuralgia subsided, the tenderness of the uterus and the ovaries entirely disappeared, and her condition was so much improved that it seemed to me that this was an efficient means of treating pelvic troubles. In the midst of this work I found that Dr. Englemann, of St. Louis, was utilizing the same idea. He was using medicated applications such as iodine, carbolic acid, sulphate of zinc, tannin, iodoform, etc., and he made this subject the text of his annual address to the Gynæcological Society of St. Louis. I think the gist of his paper was incorporated in the report I made to the State Medical Society last summer at Bloomington. Since then, in all sorts of cases of uterine inflammation, I have been making applications of the cotton tampon, resulting in considerable dissatisfaction with the material employed, and I have commenced the use of something else. It is a preparation of wool that is called "antiseptic wool." This wool is finely carded, free from all oil and foreign substances. A piece is cut off, of such a length as will fit nicely into the vagina, and then with the patient in the genu-pectoral position, with the perineum retracted, this is stuffed into the vagina and left there. The upper end of this tampon can be soaked in any antiseptic solution, as boro-glyceride or listerine, and with a piece of string attached to the lower end of it, the patient can remove it and douch the vagina, in readiness for the next tampon, and in this way tampon after tampon can be introduced and the uterus held up to the highest possible level, and advantage taken of the natural drainage from the uterus of the superabundant amount of blood. The inflammations of the uterus we are usually called upon to treat are not active, but chronic, and if we hold the uterus up so that it can drain itself properly through the veins, the nutritive changes which take place will be facilitated to the greatest extent. A small Sims' speculum can easily be applied without trouble to the patient, and this wool can be pushed into the vagina so that when the patient gets up she has a soft elastic cushion for the uterus to rest upon. In this way the greatest comfort is at once experienced. I have treated between twenty and thirty cases in this way. One case was a woman with a severe laceration of the neck of the uterus; the probe went into the cavity about 4½ inches. Local treatment had been freely employed in this case. She had pain in the legs and hips and profuse menstruation, and was a total wreck when I saw her. I put her in position and applied the tampon. I found after using four or five of them the pains had nearly all disappeared. I found also that the raw edges of the torn cervix were taking on a new mucous membrane, and I had the gratification of finding, after six months, that this uterus had been reduced to its natural size. It was with difficulty the laceration could be recognized through the speculum. I have recently been called to see a woman who has inflammation of the ovary upon the right side. I found her in bed, where she had been five months continuously. Upon moving the uterus, I found there was a great deal of tenderness throughout the pelvic tissues and around the right ovary, the slightest touch producing the greatest suffering. The woman was put in the genu-pectoral position and the vagina plugged with this wool. She got out of bed the next day, and the next night went down to dinner. Now she is going all over Chicago. Before that she had been treated nine months by means of local applications, tonics, laxatives and everything of that kind.

The result of the support of the uterus and holding the ovary up has been almost marvelous. I make these statements concerning this method of treatment for the purpose of calling attention to it, as I am still studying the subject. These tampons are removed after four or five days without the slightest odor upon them.

When the uterus is enlarged it becomes heavy, sinks, and presses the veins which carry the blood out of the uterus, and we have strangulation. By raising the uterus up, the blood flows freely and the nutritive changes tend always to health. One outgrowth of the use of this tampon may be that many cases of laceration of the cervix, now operated upon, may escape operation. I have been surprised to see how very nicely patients get along, even though they have extensive lacerations, under this treatment.

W. W. JAGGARD, M.D.

2330 Indiana Avenue.

